# Analysis of Pollution Hazard Intensity: A Spatial Epidemiology Case Study of Soil Pb Contamination

**DOI:** 10.3390/ijerph13090915

**Published:** 2016-09-14

**Authors:** Hoehun Ha, Peter A. Rogerson, James R. Olson, Daikwon Han, Ling Bian, Wanyun Shao

**Affiliations:** 1 Department of Sociology, Anthropology and Geography, Auburn University at Montgomery, 7041 Senators Drive, Montgomery, AL 36117, USA; wshao@aum.edu; 2 Department of Geography, University at Buffalo, Wilkeson Hall, Buffalo, NY 14261, USA; rogerson@buffalo.edu (P.A.R.); lbian@buffalo.edu (L.B.); 3 Departments of Pharmacology and Toxicology and Epidemiology and Environmental Health, Farber Hall, University at Buffalo, Buffalo, NY 14214, USA; jolson@buffalo.edu; 4 Department of Epidemiology and Biostatistics, School of Public Health, Texas A&M University, College Station, TX 77843, USA; dhan@sph.tamhsc.edu

**Keywords:** soil lead (Pb) contamination, physical and socioeconomic/demographic characteristics, spatial modelling, Anniston, Alabama

## Abstract

Heavy industrialization has resulted in the contamination of soil by metals from anthropogenic sources in Anniston, Alabama. This situation calls for increased public awareness of the soil contamination issue and better knowledge of the main factors contributing to the potential sources contaminating residential soil. The purpose of this spatial epidemiology research is to describe the effects of physical factors on the concentration of lead (Pb) in soil in Anniston AL, and to determine the socioeconomic and demographic characteristics of those residing in areas with higher soil contamination. Spatial regression models are used to account for spatial dependencies using these explanatory variables. After accounting for covariates and multicollinearity, results of the analysis indicate that lead concentration in soils varies markedly in the vicinity of a specific foundry (Foundry A), and that proximity to railroads explained a significant amount of spatial variation in soil lead concentration. Moreover, elevated soil lead levels were identified as a concern in industrial sites, neighborhoods with a high density of old housing, a high percentage of African American population, and a low percent of occupied housing units. The use of spatial modelling allows for better identification of significant factors that are correlated with soil lead concentrations.

## 1. Introduction

In the 1920s, Anniston was the nation’s largest producer of cast-iron soil pipe and was known as the “Soil Pipe Capital of the World”, with an annual production of about 140,000 tons [1]. Various types of chemical contaminants in foundry waste, including heavy metals, may pose a health concern [2], since they are components of many alloys widely used for casting.

Lead (Pb) tends to headline discussions over the potential risks of living in an urban environment, both because it is mobile in urban areas and because of the health risk it poses. The US Environmental Protection Agency (EPA)’s investigation of Pb contamination in Anniston began when high concentrations of lead were found in soil when sampling for polychlorinated biphenyls (PCBs) during 1999–2000. The EPA completed an investigation of commercial and residential areas in Anniston and remediated soil in sites with high levels of lead (400 mg/kg and greater). As a result, companies that presently or formerly owned the foundries in Anniston agreed to pay for residential lead clean-up in some local properties [3].

Lead in urban soils comes from several sources. In industrial areas, lead is usually attributed to atmospheric deposition from smelting or use of casting molds in local pipe foundries [4,5,6,7]. Along highways, Pb contamination is attributed to exhaust emissions [8,9]. In residential areas, most of Pb contamination is attributed to the deterioration of lead-based paint for housing [10,11,12,13]. An estimated 83% of all US houses built before 1978 still contain potentially dangerous quantities of lead and it remains on the interior and exterior walls of houses to this day [14]. One study also reports that 52% of houses built in the US before the late 1970s have yard soil Pb levels over 400 mg/kg, the EPA screening level for soil Pb contamination [15].

There are also several studies on the associations between soil lead concentrations and populations by race and income-to-poverty ratio in urban areas [16,17]. Both racial and income disparities have been documented with respect to exposure to lead in soils. Residential areas with a high prevalence of racial minorities, and individuals with a low income-to-poverty ratio, were positively associated with spatially interpolated lead concentrations, supporting the conclusion that they were disproportionately exposed to environment contaminants [17,18,19,20].

Moreover, other studies on the transport and distribution of lead in the environment emphasize the usefulness of spatial modelling [11,21,22,23,24,25,26]. These studies note that multivariate statistical and geostatistical approaches have been adopted to reduce costs for investigation, as well as to minimize and quantify uncertainties [23,26,27]. In particular, kriging utilizes the spatial autocorrelation principle to interpolate point samples to areal maps. The method has been widely adopted in environmental contamination studies such as producing regional distribution maps of nonpoint sources of heavy meatal contamination [25,28,29,30]. Compared to the levels previously observed in other urban and industrial areas, Pb concentrations in Anniston soils are significantly higher [25,28,29].

The primary objectives of this study are to describe the effects of physical variables on soil lead levels in Anniston AL, and to identify the socioeconomic and demographic characteristics of those residing disproportionately in areas with higher soil lead concentrations. Furthermore, the work aimed to use spatial regression modelling to correct for potential spatial autocorrelations and account for non-constant error variance, which ultimately allowed for better identification of significant factors associated with soil Pb contamination in the region.

## 2. Materials and Methods

### 2.1. Collection of Data

**Figure 1:**
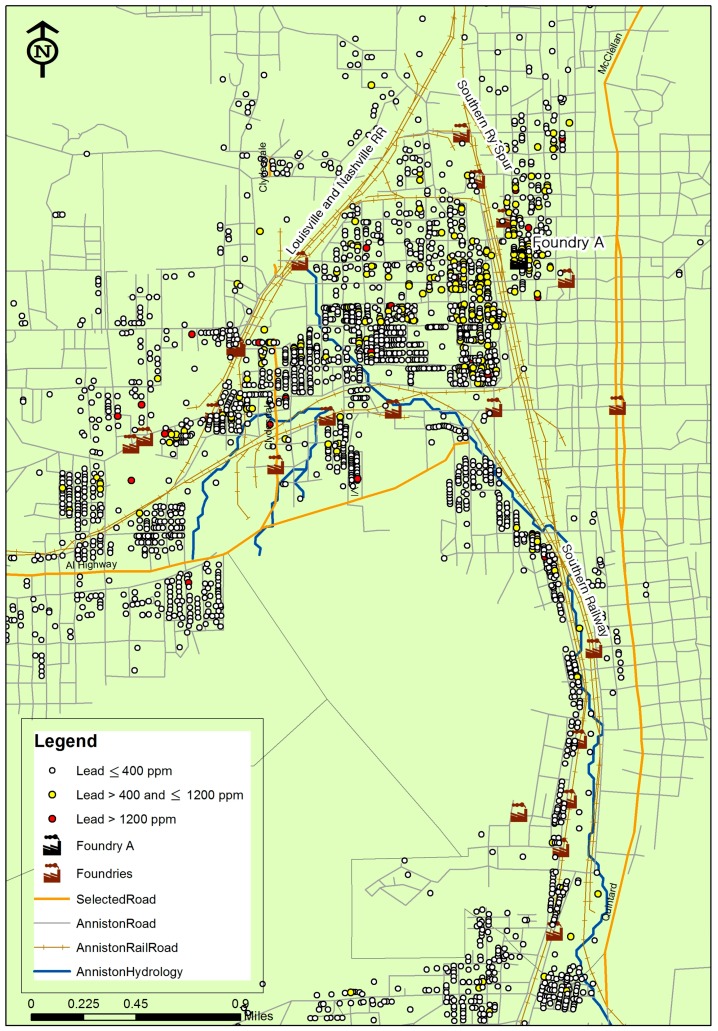
Soil lead levels at sampling locations in Anniston, AL. ppm: part per million.

Anniston, Alabama, is located approximately 60 miles east of Birmingham and 90 miles west of Atlanta. It is a community of about 23,000 people and is situated in Calhoun County. The specific areas of interest are residential neighborhoods near 23 former and currently operating foundries, highways, and major railroads in the study area, as shown in Figure 1. Information on physical variables and soil Pb levels is collected from several data sources. A database of soil samples collected from 2000 to 2008 in Anniston was obtained from the US EPA that includes measurement of Pb levels at 9365 sample locations from residential properties. Multiple lead measurements were taken from the upper 3 inches of soil in each location and reported in parts per million (ppm or mg/kg). Pb levels were measured by US EPA method 3050/6010/6020 (ICP-MS: Inductively coupled plasma-mass spectrometry) [31]. All soil samples had Pb levels above the matrix specific level of quantification, which ranged from 0.5 to 7 ppm.

The latitude and longitude coordinates contained in the database are used to convert the database into a spatial database. To produce a reliable analysis of lead concentrations, the average concentration is computed for each residential property. This process results in averaged soil lead levels at a total of 2717 residential properties.

Two data sources, Digital Elevation Model (DEM) and Soil Survey Geographic Database (SSURGO), are used to extract some of the physical variables shown in Table 1. Our initial choice of physical variables included those related to distance from 23 foundries within the study region, distance from roads (highways), distance from railroads, distance from ditches, and distance from each stream order, slope, and soil type. Streams are classified in order of first through to ninth; stream order 1 is the smallest of streams and consists of small tributaries while stream order 9 is considered a river. Slope position is divided into six categories; position 1 indicates locations high on the slope, and position 6 indicates locations near the bottom of the slope. All distance measurements are in meters and fourteen soil texture types are summarized in Table 1. Soil type is also classified into runoff categories (high, medium and low), into three soil hydrology classes (B: silt loam, C: sandy clay loam, D: clay loam) and into four drainage categories (well, somewhat well, moderate, poor). The final merged dataset that is prepared for further regression analyses contained values of these physical variables for soil sample locations.

Because we are also interested in the socioeconomic and demographic characteristics of the residents living in areas with higher soil lead concentrations, socioeconomic and demographic profiles are extracted from the US EPA and US Bureau of the Census 2010 census block (smallest geographic unit used by the US Census) and block group (a geographic unit between the Census Tract and the Census Block) level data as shown in Table 2. The Census dataset provides population counts and percentages by race, gender, age, household family, housing unit, education, and income for each of the 4358 census blocks and 87 census-block groups that had recorded populations and percentages. Age is collapsed into the following seven age groups; 0–9, 10–19, 20–29, 30–39, 40–49, 50–64 and 65+. Education level is divided into four categories: no education, elementary school education, high school education, and college or graduate school education. Similarly, the Census dataset includes household family characteristics (average household size, average family size and percent family household), housing unit (occupied housing unit, renter occupied housing unit, housing units built before 1970 and others), employment (labor force and employed labor force), and income (median income and poverty level). We then determine which census block or block-group each soil sample belonged to and then assigned its socioeconomic and demographic profiles to the soil sample for further regression analyses.

### 2.2. Regression Analyses

**Figure 2:**
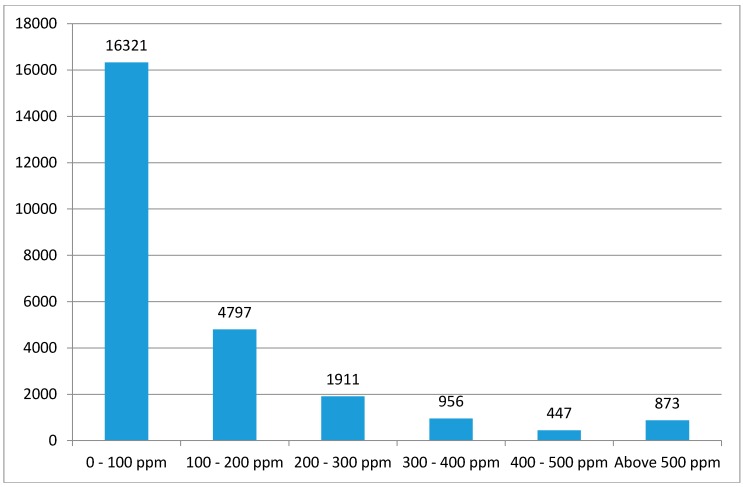
Distribution of soil Pb concentration (ppm or mg/kg).

Pollution hazard intensity is modelled at the individual soil lead sample through linear regression on physical and socioeconomic profiles identified during the review of the literature. Ordinary least squares (OLS) stepwise regressions are estimated to find independent variables that are statistically significant in each model at the 0.05 significance level for entry and 0.10 for removal. Results are further checked for multicollinearity based on the variance inflation factor (VIF), where one of the variables concerned is dropped from further consideration. Because the distribution of lead concentrations is markedly skewed, with many small values and a small number of large values as shown in Figure 2, we use a logarithmic transformation of concentration as the dependent variable. Hence our initial equation is:

(1) $$ln\left(lead\right)=b_{0}+b_{1}x_{1}+\ldots$$

where lead is the observed concentration in mg/kg, and the *x*’s represent independent variables, while the *b*’s represent coefficients for each independent variable.

Given the spatial nature of the data, spatial autocorrelation and heteroscedasticity need to be tested since the residuals and the dependent variables may exhibit not only spatial dependencies but also non-constant error variance. Spatial dependency is a situation where the error term or the dependent variable at a location is correlated with observations on the dependent variable at other nearby locations. Diagnostics of spatial dependencies in the dependent variable and in the residuals are evaluated in the statistical software GeoDaSpace to account for potential spatial effects that may affect estimation results obtained from OLS regressions [31,32,33]. Specifically, the Lagrange Multiplier (LM) test pertaining to both the spatial lag (LM lag) and spatial error models (LM error) are calculated. If both tests are statistically significant, the robust form of the test is used to determine the appropriate model.

Spatial effects evidenced by autocorrelation can be handled econometrically in two primary ways. The spatial lag model includes a spatially lagged dependent variables, *Wy*, as one of the explanatory variables:

(2) y=ρWy+Xβ+ε

where **y** is a column vector containing the dependent variable; **X** is a matrix with a column of ones representing the intercept followed by columns that represent the independent variables, **β** is a vector of regression coefficients, **ε** is a vector of random error terms, and **ρ** is a spatial autoregressive coefficient. **W** is a *n* × *n* matrix containing zeros on the diagonal; the off-diagonal elements *w_ij_* indicate the strength of the relationship between location *i* and location *j*. In all of the spatial models estimated for this study, the spatial weights matrix is specified using a threshold distance criterion. This is the minimum distance required to ensure that each location has at least one neighbor.

The spatial error model expresses each residual as a function of surrounding residuals. The spatial error model is given by:

(3) y=xβ+ε

where

(4) ε=λWε+ξ

with the same notation as above and where **λ** is an autoregressive regression coefficient, and **Wε** captures the spatial lag for errors and the elements of **ξ** are normally distributed with mean 1 and variance 1. A spatial error model is estimated by maximum likelihood, while a spatial lag model is best estimated by a two-stage least-squares (2SLS) procedure, which does not assume normality of errors and can accommodate a correction for heteroscedasticity, if present [32,33,34].

A proper regression model is selected by GeoDa’s guidelines [33]. If the OLS regression exhibits heteroscedasticity only, as shown by the Breusch-Pagan test, then the White correction is applied to the OLS results [35]. However, if the results of the LM-lag test are significant (or more significant than the LM-error test), then the spatial lag model is carried out as the alternative regression. After the model is run, we apply the Anselin-Keleiian test for residual spatial autocorrelation. If the results of the latter are significant, the model is re-estimated with the heteroskedastic and autocorrelation robust (HAC) approach of Kelejian and Prucha [35]. In the case where the LM-error test is significant (or more significant than the LM-lag test), then a spatial error model is estimated; in case of heteroscedasticity, the Kelejian-Prucha consistent estimator for heteroscedastic error terms (KP-HET) is used [34,36,37].

## 3. Results

Two regression models of contamination intensity on soil lead are presented here. Contamination intensities are modelled with physical and socioeconomic profiles, and estimated results are summarized in Table 3, Table 4, Table 5 and Table 6.

### 3.1. Spatial Regression Results: Physical Variables

The Breusch-Pagan test and LM test show that the model for physical variables displays both heteroscedasticity and spatial dependency. Also, the LM-error test is more significant than the LM-lag test:

Breusch-Pagan test = 130.241 (*p* < 0.001)
Robust LM (lag) = 6.295 (*p* = 0.012)
Robust LM (error) = 665.910 (*p* < 0.001)

Following the procedure outlined earlier for selecting a spatial regression model, a spatial error model is estimated and reported in Table 3. This model has an explanatory power of *R*^2^ = 0.372 and thirteen physical variables from the initial OLS regression, after accounting for covariates and multicollinearity, are selected to represent the physical profiles which are hypothesized to play a significant role in explaining soil lead concentrations. The distance to a specific foundry (Foundry A) is a significant factor in soil Pb contamination. Similarly, distance to railroad is captured in the model as a contributing risk factor. Signs of the effects are in the expected direction; higher soil lead concentrations are likely to be identified in soil samples that are collected at distances closer to Foundry A and railroads, which represent potential point sources for lead in the region.

In addition to these potential Pb contamination sources, two other physical profiles, soil texture class, particularly gravelly loam and elevation, are found to be statistically significant factors of soil contamination intensity. Specifically, gravelly loam soil is found to have positive influences on soil contamination intensity, whereas elevation is negatively associated with the intensity. Thus, higher soil Pb levels are more likely to be found in residential properties covered with gravelly loam, but are less likely to be found in high elevation areas. Moreover, the coefficients for the slope position and soil drainage class variables are all negative (relative to the omitted categories, which are the flat slope position and poor drainage), implying that locations with a higher slope and good drainage soil conditions tend to have lower lead concentrations.

The inclusion of the spatial error term reduces the absolute magnitude of many of the coefficients, as well as their statistical significance as shown in Table 4. In addition to the spatial error term, this spatial error model allows us to correct problems of heteroscedasticity, which potentially could lead to the inclusion of insignificant variables in the model. In the results, distance to highways, distance to stream order 6, and soil hydrology class B became insignificant after correcting for spatial dependency and heteroscedasticity in the model. This implicates that the inclusion of these variables can now be attributed to the spatial autocorrelation and non-constant error variance. The other variables that remain in the equation, however, are highly significant. The results in Table 4 demonstrate an important and well-known issue with regression using spatial data: failure to account for spatial effects can make independent variables seem more significant than they actually are.

### 3.2. Spatial Regression Results: Socioeconomic Variables

The model of Pb contamination for socioeconomic profiles shows both heteroscedasticity and spatial dependency as indicated by the following diagnostic tests:

Breusch-Pagan test = 180.326 (*p* < 0.001)
Robust LM (lag) = 64.630 (*p* = 0.001)
Robust LM (error) = 1166.421 (*p* < 0.001)

On this basis, estimates of a spatial error model of soil Pb levels for socioeconomic variables are presented in Table 5. The initial OLS model has a goodness-of-fit of *R*^2^ = 0.288. This model reveals that soil lead concentrations are significantly higher in the regions with old housing and where predominantly low-income and African American populations reside. This model further documents the uneven distribution of soil lead contamination across the city landscape, which disproportionately impacts populations with a low level of education. Furthermore, Pb levels in the residential soils collected in areas with a larger average family size are significantly higher than those in residential soils sampled from areas with a smaller family size. In addition, two housing unit variables, namely the percent of occupied housing units, and percent of owner occupied housing units, are found to be statistically significant profiles that are negatively associated with pollution hazard intensity patterns in the region.

These results are all consistent with the observation that the higher residential soil Pb levels are more likely to be found in regions with older homes, a low percent of occupied housing units, and in residential neighborhoods that have a disproportionately higher number of minorities and older residents.

In the spatial error model shown in Table 6, it is noteworthy that the value of *R*^2^ dropped to 0.276. Six socioeconomic variables become insignificant, including the most significantly associated factors captured in the initial OLS model (percent of high school degrees received, percent of population from 50 to 64 years of age, female median age, percent of single parents, poverty level, and percent of owner housing units). All of the remaining five socioeconomic variables, namely percent of housing units built before 1970, percent of no education received, percent of African Americans, average family size, and percent of occupied housing units, remain in the model with slightly reduced absolute magnitude of the coefficients and their statistical significance.

## 4. Discussion

Anniston AL has had a long history of heavy industrial operations that contributed, in part, to lead contamination in residential soil. This study attempts to answer fundamental questions, starting with characterizing the magnitude and spatial distribution of residential soil Pb contamination across the region and identifying important point or non-point sources of the contamination. An additional related aim of the study was to identify important residential characteristics associated with the spatial patterns of soil Pb in Anniston. In order to address these questions, this study has analyzed the physical and socioeconomic profiles associated with soil Pb contamination in this industrial urban environment.

### 4.1. Implications for Spatial Regression Results: Physical Variables

In the model with physical profiles, we identify distance to a specific foundry, namely Foundry A and distance to railroads as important explanatory variables affecting soil Pb concentrations after adjusting for covariates and multicollinearity.

Soil Pb contamination in the study area may possibly be caused by solid waste and air emissions generated during years of operations of local foundries. Foundry A, as shown in Figure 1, has been in operation since 1917, and is known as a major pipe manufacturer in the study area, using molds made out of sand to make the cast-iron pipe. Large quantities of lead contaminated sand from the foundry would contaminate the area, in addition to the relocation of contaminated sand to yards and gardens of Anniston residents [38]. Foundry A is involved in the production of commercial, plumbing, industrial, and marine products for the US Navy. Based on EPA reports, waste sand from Foundry A contains up to about 3000 ppm of lead, and approximately 600 ppm is typically dissolved into soils [38].

Changes in Pb concentrations in the study area are closely related to the distance to the potential pollution source, where each 100 m increase in distance from Foundry A corresponds to a decrease of 2.65% in Pb levels in soils. This pattern is probably due to the fact that soil in the proximity of the foundry can have a greater influence on Pb exposures by disposals of heavy metals to the ground and air from the foundry, while locations further from the foundry have a less direct impact on Pb exposures through the foundry. It is important to note that this analysis can be validated by the fact that Foundry A has been recorded as the largest Pb disposal facility in the study area based upon information from the EPA Toxic Release Inventory (TRI) website [39]. Most of the other former and current foundries, with the exception of Foundry A and one other foundry in the area, have never reported releasing Pb into the environment [7,39].

Coal burning and diesel exhaust from railroads are also well-known as a common source of soil lead contamination [23,40,41]. It is interesting to note that in the model, even after controlling for other potential Pb sources (e.g., proximity to the roads), distance to railroads remains a significant factor negatively associated with Pb concentration. That is, each 100 m increase in the distance from the railroads leads to a 3.6% decrease of Pb level in soils. As shown in Figure 1, there is one major railroad (the Southern Railway) passing through the regions with high Pb levels, implying that Pb exposures in soils would be relatively high in close proximity to the major railroad. The Southern Railway is known as having the longest continuous line of railway in US, and this historic railroad operation may have resulted in the presence of contamination reported today. The most commonly reported contamination along railroads includes heavy metals (such as lead and arsenic) and constituents of oil or fuel. These chemicals have been associated with normal railroad operations and are likely to be found anywhere along the line [42,43].

### 4.2. Implications for Spatial Regression Results: Socioeconomic Variables

This study also focuses on the characterization of socioeconomic profiles in the residential properties that are associated with high soil Pb levels. It attempts to integrate socioeconomic profiles with geographic variation of soil Pb contamination to evaluate whether high levels of soil Pb are clustered and whether discriminating distribution patterns exists among different socioeconomic profiles. We observe five socioeconomic variables including the percent of housing units built before 1970, percent of no education received, percent of African Americans, average family size, and percent of occupied housing units to be significant factors associated with elevated soil Pb levels, which increase the risk of human exposure to Pb. One of our initial interpretations is that in industrial landscapes and in neighborhoods with high densities of old housing and a low percentage of occupied housing units, the risk of Pb contamination at such sites is high. Our results are supported in part by several studies concluding that in residential areas, most Pb contamination is attributed to the paint used for old housing [10,11,12,13]. In relation to the association found with soil lead contamination and a low percentage of occupied housing units, other studies have found that there may be approximately a half-million vacant and abandoned residential properties across the nation, including brownfields and idle former industrial properties with real or perceived environmental contamination [44].

Racial disparities are also observed in the current study with respect to the association between block group race/ethnicity characteristics and soil Pb contamination. In general, a greater number of blacks by block group is positively associated with soil Pb concentrations in the study area. That is, there are many soil samples that have high Pb levels, and these are distributed in the census blocks where a high percentage of the African Americans with a high poverty level (*β* = 0.442, *p* < 0.001) resides. These environmentally impacted neighborhoods are relatively close to the potential sources of the contamination such as foundries and railroads [14,17,45]. A reasonable interpretation would be that economically disadvantaged people cannot be as selective of where they live, so they often are more likely to live near environmentally impacted neighborhoods, near heavy industry and railroads. Other related studies have also found that minority populations often reside in neighborhoods impacted by a range of environmental health concerns. In New Orleans, LA, USA, Campanella and Mielke found that as soil Pb concentrations increase, the percentage of the African Americans also increases; African-Americans are also more than two times as likely as non-African-Americans to live where Pb soil concentrations are >100 mg/kg [46]. Furthermore, Zaharan et al. found a significant positive association between the percentage of African-American children at a school and median blood Pb concentrations [45]. Overall, the extensive database of soil lead levels in Anniston, AL allowed our study to model the socioeconomic characteristics of those residing disproportionately in areas with higher soil Pb concentrations. Hence, our findings have important policy implications in further understanding the disparate burden of soil Pb exposure in Anniston and potentially other communities with a similar history of industrial activity.

### 4.3. Limitations and Future Studies

There may have been some bias in our studies since we have used two different geographic levels of data for the explanatory variables, especially in the model with socioeconomic profiles. We have aggregated data at the level of the census block or block-group for our explanatory variables, whereas individual soil location is used for the dependent variable. Thus, the exact information on socioeconomic profiles corresponding to each soil sample site is not available, implying that there might be an issue of ecological fallacy, since analyses based on aggregated data could lead to conclusions different from those based on individual data.

Based on our findings, it will be of great interest in the future to examine the comparison of these measures of soil Pb levels with serum Pb levels in individuals living in the study area. These regression models can be used to estimate soil Pb concentrations at locations where no measurements are obtained. In turn, these models will ultimately serve as an input in assessing whether soil Pb levels estimated at residential locations can be a possible surrogate for actual serum lead concentrations in individuals. In the next stage of this research, predicted soil Pb levels generated from the model will be compared with serum Pb levels in individuals living in the study area to determine correlations between soil and serum Pb level [14,17,45].

## 5. Conclusions

The concentration and spatial distribution of soil Pb contamination in relation to potential industrial point sources of pollution in Anniston AL were thoroughly investigated in this study. A major focus of this study was to examine physical characteristics associated with the magnitude of soil Pb contamination, as well as to identify the socioeconomic profiles of those residing disproportionately in areas with higher soil lead contamination.

This case study tried to integrate complex data on the geographic variation of soil Pb levels to assess whether soil Pb contamination is clustered and whether discriminating distribution patterns exist between different physical factors and between socioeconomic profiles. Regression analysis is carried out to identify important factors associated with Pb concentrations. Two different regression models (physical model and socioeconomic model) are created and in the physical model, soil Pb levels are identified to be significantly influenced by the proximity to the potential sources of Pb contamination, namely Foundry A and major railroads. In addition, in the socioeconomic model, the study has found that residential areas with a high percent of old houses, a low percent of occupied housing units and high percentage of racial/ethnic minorities, are positively associated with soil Pb concentrations.

Overall, this study allows local communities to comprehend more precisely where high Pb contaminations are occurring, and these analyses facilitate understanding of the associated factors associated with soil Pb contamination in the region. This evidence-based information is necessary to draw public attention and to identify areas with high soil Pb contaminations for further interventions, including clean-up programs, to effectively remediate lead contamination in areas with high soil Pb levels. Our findings have important public health and policy implications in further understanding the disparate burden of soil Pb exposure in Anniston, AL and potentially other communities with a similar history of industrial activity.

## Figures and Tables

**Table 1:** List of physical variables used for the regression models.

Variable	Data Source	Description
Distance to each foundry	EPA:TRI	unit: meters
Distance to roads (highways)	Census TIGER	unit: meters
Distance to railroads	Scanning	unit: meters
Distance to ditches	Scanning + census tiger	unit: meters
Distance to each stream order (stream order 1 through stream order 9)	DEM	unit: meters (stream order 1 being the smallest of streams and stream order 9 being considered a river)
Elevation	DEM	unit: meters
Slope position	DEM	Categorical value 1: Ridge—(Slope position 1) 2: Upper Slope—(Slope position 2) 3: Middle Slope—(Slope position 3) 4: Flat Slope—none 5: Lower Slope—Slope position 4) 6: Valley—(Slope position 5)
Soil Runoff class	SSURGO	4 types: High, Medium, Low, Negligible
Soil Drainage class	SSURGO	4 types: Well, Somewhat well, Moderately, Poorly
Soil Hydrology class	SSURGO	3 types: B (silt loam or loam), C (sandy clay loam), D (clay loam)
Soil Texture Class	SSURGO	15 types CB-FSL: cobbly fine sandy loam CL: clay loam CN-L: channery loam CR-SIL: cherty silt loam FSL: fine sandy loam GR-CL: gravelly clay loam GR-FSL: gravelly fine sandy loam GR-L: gravelly loam GR-SCL: gravelly sandy clay loam GR-SIL: gravelly silt loam SIL: silt loam L: loam ST-FSL: stony fine sandy loam ST-L: stony loam ST-SL: stony sandy loam

EPA: Environmental Protection Agency. TRI: Toxic Release and Inventory, a resource for learning about toxic chemical releases and pollution prevention activities reported by industrial and federal facilities. DEM: Digital Elevation Model. SSURGO: Soil Survey Geographic database refers to digital soils data produced by the Natural Resources Conservation Service.

**Table 2:** List of socioeconomic variables used for the regression models.

Category		Variable	Data Source
Socioeconomic Variables	Race	Percent African American	Census Block
	Gender	Percent female	Census Block
	Population	Percent of population to 9 years	Census Block
		Percent of population 10 to 19 years	Census Block
		Percent of population 20 to 29 years	Census Block
		Percent of population 30 to 39 years	Census Block
		Percent of population 40 to 49 years	Census Block
		Percent of population 50 to 64 years	Census Block
		Percent of population over 65 years	Census Block
	Age	Median age	Census Block
	Household	Average household Size	Census Block
	Family	Average family size	Census Block
		Percent family household	Census Block
	Housing unit	Occupied housing unit	Census Block
		Owner occupied housing unit	Census Block
		Renter occupied housing unit	Census Block
		Percent single mother	Census Block
		Percent single father	Census Block
		Percent single parent	Census Block
		Percent of housing units built before 1970	Census Block Group
	Education	Percent no education	Census Block Group
		Percent elementary school education	Census Block Group
		Percent high school education	Census Block Group
		Percent college or graduate school education	Census Block Group
	Employment	Percent labor force	Census Block Group
		Percent of labor force employed	Census Block Group
	Income	Median income	Census Block Group
		Percent Poverty poverty Levellevel	Census Block Group

**Table 3:** Estimated results of regression models of pollution hazard intensity, as a function of physical profiles; Ordinary least squares (OLS) regression.

*Model 1. OLS Regression Model for Physical Variables- R^2^ = 0.380*			
**Variable**	**Beta**	***t*-Value**	***p*-Value**
Constant	7.604	24.885	<0.001
Distance to Foundry A ***	−2.54 × 10^−4^	−18.182	<0.001
Distance to railroads ***	−4.61 × 10^−4^	−8.363	<0.001
Gravelly loam ***	0.353	7.062	<0.001
Distance to 4th stream order ***	5.68 × 10^−4^	4.714	<0.001
Elevation ***	−7.94 × 10^−3^	−6.269	<0.001
Slope position 5 (Valley) ***	−0.409	−4.272	<0.001
Somewhat well soil drainage	−0.360	−5.900	<0.001
Well soil drainage ***	−0.446	−6.223	<0.001
Slop position 4 (Lower slope) ***	−0.116	−3.441	<0.001
Soil hydrology class B (Silt) ***	0.167	3.517	<0.001
Distance to roads (highways) *	−1.76 × 10^−3^	−2.294	0.021
Distance to 6th stream order **	9.91 × 10^−5^	2.735	0.006
Low soil run-off *	−0.094	−2.101	0.035

* *p* < 0.05 (one-tail test); ** *p* < 0.01 (one-tail test); *** *p* < 0.001 (one-tail test).

**Table 4:** Estimated results of regression models of pollution hazard intensity, as a function of physical profiles; Spatial error regression.

*Model 2. Spatial Error Regression Model for Physical Variables- R^2^ = 0.372*			
**Variable**	**Beta**	***z*-Value**	***p*-Value**
Constant	7.985	11.560	<0.001
Distance to Foundry A ***	−2.65 × 10^-4^	−7.614	<0.001
Distance to railroads *	−3.60 × 10^−4^	−2.222	0.026
Gravelly loam ***	0.350	5.856	<0.001
Distance to 4th stream order *	4.37 × 10^−4^	2.293	0.021
Elevation ***	−9.82 × 10^−3^	−3.202	0.001
Slope position 5 (Valley) **	−0.268	−2.613	0.008
Somewhat well soil drainage **	−0.157	−2.348	0.018
Well soil drainage ***	−0.288	−3.445	<0.001
Slop position 4 (Lower slope) **	−0.095	−2.714	0.006
Soil hydrology class B (Silt)	0.069	1.199	0.230
Distance to roads (highways)	−1.51 × 10^−3^	−1.639	0.101
Distance to 6th stream order	1.00 × 10^−4^	1.210	0.226
Low soil run-off ***	−0.171	−3.665	<0.001
Lamda	0.733	15.707	<0.001

* *p* < 0.05 (one-tail test); ** *p* < 0.01 (one-tail test); *** *p* < 0.001 (one-tail test).

**Table 5:** Estimated results of regression models of pollution hazard intensity, as a function of socioeconomic profiles; OLS regression.

*Model 3. OLS Regression Model for Socioeconomic Variables- R^2^ = 0.288*			
**Variable**	**Beta**	***t*-Value**	***p*-Value**
Constant	3.592	21.902	<0.001
% of Housing unit built before 1970 ***	0.025	16.055	<0.001
% of No education received ***	0.112	8.445	<0.001
% African American ***	4.76 × 10^−3^	7.372	<0.001
% of High school degree received *	−0.011	−2.563	0.010
Average family size ***	0.123	6.794	<0.001
% of Population 50 to 64 years ***	4.85 × 10^−3^	3.816	<0.001
% of Occupied housing unit ***	−4.39 × 10^−3^	−4.709	<0.001
Female median age **	3.32 × 10^−3^	2.855	0.004
% of Single parent **	1.21 × 10^−3^	3.286	0.001
% of Poverty level ***	6.51 × 10^−3^	3.521	<0.001
% of Owner occupied housing unit **	−4.64 × 10^−3^	−3.82	0.001

* *p* < 0.05 (one-tail test); ** *p* < 0.01 (one-tail test); *** *p* < 0.001 (one-tail test).

**Table 6:** Estimated results of regression models of pollution hazard intensity, as a function of socioeconomic profiles; Spatial error regression.

*Model 4. Spatial Error Regression Model for Socioeconomic Variables- R^2^ = 0.276*			
**Variable**	**Beta**	***z*-Value**	***p*-Value**
Constant	3.928	8.207	<0.001
% of Housing unit built before 1970 ***	0.015	3.327	<0.001
% of No education received *	0.055	1.983	0.047
% African American **	1.28 × 10^−3^	2.853	0.004
% of High school degree received	−5.88 × 10^−3^	−0.632	0.526
Average family size ***	0.064	3.314	<0.001
% of Population 50 to 64 years	1.22 × 10^−3^	1.002	0.316
% of Occupied housing unit *	−2.04 × 10^−3^	−1.974	0.048
Female median age	1.63 × 10^−3^	1.283	0.199
% of Single parent	1.54 × 10^−4^	0.460	0.645
% of Poverty level	3.57 × 10^−3^	0.873	0.382
% of Owner occupied housing unit	−1.22 × 10^−4^	−0.031	0.975
Lamda	0.860	24.939	<0.001

* *p* < 0.05 (one-tail test); ** *p* < 0.01 (one-tail test); *** *p* < 0.001 (one-tail test).
